# Supplementary material for: Knowledge of female genital schistosomiasis and urinary schistosomiasis among final-year midwifery students in the Volta Region of Ghana
Source: PLoS One. 2024 May 2;19(5):e0302554. doi: 10.1371/journal.pone.0302554 (PMC11065279; doi:10.1371/journal.pone.0302554)
Supplement: S2 Checklist — (DOCX) [file pone.0302554.s002.docx]

STROBE Statement—checklist of items that should be included in reports of observational studies

|  | Item No. | Recommendation | Page  No. | Relevant text from manuscript |
| --- | --- | --- | --- | --- |
| **Title and abstract** | 1 | (*a*) Indicate the study’s design with a commonly used term in the title or the abstract | 2 | A cross-sectional study was conducted among 193 randomly selected final-year students from all three midwifery training institutions in the Volta Region of Ghana |
|  |  | (*b*) Provide in the abstract an informative and balanced summary of what was done and what was found | 2/3 |  |
| Introduction | | | |  |
| Background/rationale | 2 | Explain the scientific background and rationale for the investigation being reported | 5/6 | Final year midwifery students in Ghana constitute a pre-service group who, upon completion of their programme of study in the various nursing and midwifery training colleges, will add on to the workforce at the various hospitals, clinics /health centers, and community-based health planning and services (CHPS) compounds to deliver primary health care (PHC). The CHPS compounds at the community level and health centers at the sub-district level form the basic levels of the health system in Ghana [21]. In most communities, the midwives serve as the first point of call either through the CHPS compounds or the clinics. If this cadre of health workers are aware of FGS, they can at least suspect it as a diagnosis and make a timely referral for the patients to be diagnosed on time and thus preventing long term complications. There is no data on the knowledge of these pre-service midwives in Ghana about FGS. To help fill this gap, the study sought to assess the knowledge and awareness of female genital schistosomiasis and urinary schistosomiasis among final year midwifery students in the Volta region of Ghana. |
| Objectives | 3 | State specific objectives, including any prespecified hypotheses 6 |  | To help fill this gap, the study sought to assess the knowledge of female genital schistosomiasis and urinary schistosomiasis among final year midwifery students in the Volta region of Ghana |
| Methods | | | |  |
| Study design | 4 | Present key elements of study design early in the paper 6 |  | The first statement under the description of the study design and area description is as follows;  “This was a cross-sectional study carried out from 22^nd^ August, 2022 to 12^th^ September, 2022 in all three midwifery training institutions in the Volta region of Ghana”……. |
| Setting | 5 | Describe the setting, locations, and relevant dates, including periods of recruitment, exposure, follow-up, and data collection | 6/7 |  |
| Participants | 6 | (*a*) *Cohort study*—Give the eligibility criteria, and the sources and methods of selection of participants. Describe methods of follow-up  *Case-control study*—Give the eligibility criteria, and the sources and methods of case ascertainment and control selection. Give the rationale for the choice of cases and controls  *Cross-sectional study*—Give the eligibility criteria, and the sources and methods of selection of participants | 8 | All final-year midwifery students in the Volta region were included in the study. Those who were below 18 years of age were excluded from the study as they were minors and required consent from their parents / guardians. This would have been operationally difficult to obtain. Students who were not available on the day of the survey were also excluded from the study. |
|  |  | (*b*) *Cohort study*—For matched studies, give matching criteria and number of exposed and unexposed  *Case-control study*—For matched studies, give matching criteria and the number of controls per case |  |  |
| Variables | 7 | Clearly define all outcomes, exposures, predictors, potential confounders, and effect modifiers. Give diagnostic criteria, if applicable | 9 |  |
| Data sources/ measurement | 8* | For each variable of interest, give sources of data and details of methods of assessment (measurement). Describe comparability of assessment methods if there is more than one group | 9 | The structured questionnaire used for data collection (see S1 Questionnaire) was developed using the information contained in a teaching manual for urinary schistosomiasis and FGS [23] as a guide. Data was collected on knowledge and awareness of urinary schistosomiasis and female genital schistosomiasis. Specifically, the questionnaire assessed mainly participants’ demographics, awareness, knowledge of signs and symptoms, complications, transmission, treatment and the prevention of urinary schistosomiasis and FGS. The questionnaire was divided into three main parts. The first part assessed the demographic characteristics of participants which included age and institution of study. The second part assessed the awareness, knowledge of signs and symptoms, complications, transmission, treatment and the prevention of urinary schistosomiasis by close-ended questions. The third part also assessed awareness, knowledge of signs and symptoms, complications, transmission, treatment and the prevention of female genital schistosomiasis by close-ended questions |
| Bias | 9 | Describe any efforts to address potential sources of bias 9 |  |  |
| Study size | 10 | Explain how the study size was arrived at 8 |  |  |

Continued on next page

| Quantitative variables | 11 | Explain how quantitative variables were handled in the analyses. If applicable, describe which groupings were chosen and why | 9 |  |
| --- | --- | --- | --- | --- |
| Statistical methods | 12 | (*a*) Describe all statistical methods, including those used to control for confounding | 9 | …..Descriptive statistics were done and presented as frequencies, proportions and percentages in tables. Chi-square test was used to assess for association between the independent variables ‘school attended’ and ‘age group’ and the dependent variables ‘ever heard of Urinary Schistosomiasis’ and ‘ever heard of FGS’. An association was said to be statistically significant if p < 0.05 |
|  |  | (*b*) Describe any methods used to examine subgroups and interactions |  |  |
|  |  | (*c*) Explain how missing data were addressed |  |  |
|  |  | (*d*) *Cohort study*—If applicable, explain how loss to follow-up was addressed  *Case-control study*—If applicable, explain how matching of cases and controls was addressed  *Cross-sectional study*—If applicable, describe analytical methods taking account of sampling strategy |  |  |
|  |  | (*e*) Describe any sensitivity analyses |  |  |
| Results | | | | |
| Participants | 13* | (a) Report numbers of individuals at each stage of study—eg numbers potentially eligible, examined for eligibility, confirmed eligible, included in the study, completing follow-up, and analysed |  |  |
|  |  | (b) Give reasons for non-participation at each stage |  |  |
|  |  | (c) Consider use of a flow diagram |  |  |
| Descriptive data | 14* | (a) Give characteristics of study participants (eg demographic, clinical, social) and information on exposures and potential confounders |  |  |
|  |  | (b) Indicate number of participants with missing data for each variable of interest |  |  |
|  |  | (c) *Cohort study*—Summarise follow-up time (eg, average and total amount) |  |  |
| Outcome data | 15* | *Cohort study*—Report numbers of outcome events or summary measures over time |  |  |
|  |  | *Case-control study—*Report numbers in each exposure category, or summary measures of exposure |  |  |
|  |  | *Cross-sectional study—*Report numbers of outcome events or summary measures | 11/12, 14-17 |  |
| Main results | 16 | (*a*) Give unadjusted estimates and, if applicable, confounder-adjusted estimates and their precision (eg, 95% confidence interval). Make clear which confounders were adjusted for and why they were included |  |  |
|  |  | (*b*) Report category boundaries when continuous variables were categorized |  |  |
|  |  | (*c*) If relevant, consider translating estimates of relative risk into absolute risk for a meaningful time period |  |  |

Continued on next page

| Other analyses | 17 | Report other analyses done—eg analyses of subgroups and interactions, and sensitivity analyses |  |  |
| --- | --- | --- | --- | --- |
| Discussion | | | | |
| Key results | 18 | Summarise key results with reference to study objectives 17 |  | To help fill existing knowledge gaps, this study assessed the knowledge of final-year midwifery students in the Volta Region of Ghana with respect to the signs and symptoms of FGS, its complications, treatment and prevention. Approximately 82% and 77% of respondents who reported hearing about FGS previously correctly selected pain during or after sex and vaginal discharge respectively as presenting symptoms. A little over 60% of respondents who reported hearing about FGS previously indicated protected sex with condoms as a preventive measure. Similarly, 50% of this population also indicated the use of vaccines as a preventive measure against FGS. While only a little over half of respondents who reported hearing about FGS correctly indicated Praziquantel as the drug for treatment, about 91% indicated antibiotics as the treatment of choice |
| Limitations | 19 | Discuss limitations of the study, taking into account sources of potential bias or imprecision. Discuss both direction and magnitude of any potential bias | 25 | The study was conducted in only the Volta Region of Ghana and the findings may be of limited generalizability. However, it may be also argued that the findings, especially those relating to FGS, will likely be similar in other regions since midwifery training institutions all over the country run the same curriculum accredited by the Ghana Tertiary Education Commission and the Nursing and Midwifery Council. The study is also limited by the use of ‘straight-jacket’ responses. A qualitative approach would have provided nuanced contexts surrounding the choice of wrong responses especially. Nevertheless, the study findings raise concerns about the poor knowledge/awareness of FGS among the study participants and constitute a reawakening call to increase efforts at making pre-service health workers know more about FGS. |
| Interpretation | 20 | Give a cautious overall interpretation of results considering objectives, limitations, multiplicity of analyses, results from similar studies, and other relevant evidence | 17-25 |  |
| Generalisability | 21 | Discuss the generalisability (external validity) of the study results | 25 | The study was conducted in only the Volta Region of Ghana and the findings may be of limited generalizability. However, it may be also argued that the findings, especially those relating to FGS, will likely be similar in other regions since midwifery training institutions all over the country run the same curriculum accredited by the Ghana Tertiary Education Commission and the Nursing and Midwifery Council. |
| Other information | |  | | |
| Funding | 22 | Give the source of funding and the role of the funders for the present study and, if applicable, for the original study on which the present article is based |  | Not Applicable |

*Give information separately for cases and controls in case-control studies and, if applicable, for exposed and unexposed groups in cohort and cross-sectional studies.

**Note:** An Explanation and Elaboration article discusses each checklist item and gives methodological background and published examples of transparent reporting. The STROBE checklist is best used in conjunction with this article (freely available on the Web sites of PLoS Medicine at http://www.plosmedicine.org/, Annals of Internal Medicine at http://www.annals.org/, and Epidemiology at http://www.epidem.com/). Information on the STROBE Initiative is available at www.strobe-statement.org.
