# Supplementary material for: Knowledge of female genital schistosomiasis and urinary schistosomiasis among final-year midwifery students in the Volta Region of Ghana
Source: PLoS One. 2024 May 2;19(5):e0302554. doi: 10.1371/journal.pone.0302554 (PMC11065279; doi:10.1371/journal.pone.0302554)
Supplement: S1 Questionnaire — (DOCX) [file pone.0302554.s003.docx]

| PART1 | | | | | | | | | | | | | | | | | | | | | | | | | | | | | | | | | | | | | | | | | | | | | | |  |  |  |
| --- | --- | --- | --- | --- | --- | --- | --- | --- | --- | --- | --- | --- | --- | --- | --- | --- | --- | --- | --- | --- | --- | --- | --- | --- | --- | --- | --- | --- | --- | --- | --- | --- | --- | --- | --- | --- | --- | --- | --- | --- | --- | --- | --- | --- | --- | --- | --- | --- | --- |
| **DEMOGRAPHIC INFORMATION** | | | | | | | | | | | | | | | | | | | | | | | | | | | | | | | | | | | | | | | | | | | | | | |  |  |  |
| Write the answer to the item where indicated or tick the appropriate response where possible | | | | | | | | | | | | | | | | | | | | | | | | | | | | | | | | | | | | | | | | | | | | | | |  |  |  |
| 1 | | | | | | | | Code | | |  | | | | | | | | | | | | | | | | | | | | | | | | | | | | | | | | | | | |  |  |  |
| 2 | | | | | | | | Age groups (in years) | | | 18-22(years) | | | | | | | | | | | | | | | | | | | | | |  | | | | | | | | | | | | | |  |  |  |
|  |  |  |  |  |  |  |  |  |  |  | 23-27(years) | | | | | | | | | | | | | | | | | | | | | |  | | | | | | | | | | | | | |  |  |  |
|  |  |  |  |  |  |  |  |  |  |  | 28-32(years) | | | | | | | | | | | | | | | | | | | | | |  | | | | | | | | | | | | | |  |  |  |
|  |  |  |  |  |  |  |  |  |  |  | 33 and above(years) | | | | | | | | | | | | | | | | | | | | | |  | | | | | | | | | | | | | |  |  |  |
| 3 | | | | | | | | School | | UHAS | | | | | | | | | | | | | | | | | | | | | | |  | | | | | | | | | | | | | |  |  |  |
|  |  |  |  |  |  |  |  |  |  | Hohoe-NMTC | | | | | | | | | | | | | | | | | | | | | | |  | | | | | | | | | | | | | |  |  |  |
|  |  |  |  |  |  |  |  |  |  | Keta-NMTC | | | | | | | | | | | | | | | | | | | | | | |  | | | | | | | | | | | | | |  |  |  |
| PART 2 | | | | | | | | | | | | | | | | | | | | | | | | | | | | | | | | | | | | | | | | | | | | | | | |  |  |
| **AWARENESS OF URINARY SCHISTOSOMIASIS** | | | | | | | | | | | | | | | | | | | | | | | | | | | | | | | | | | | | | | | | | | | | | | |  |  |  |
| In this section, answer by ticking the right answer. | | | | | | | | | | | | | | | | | | | | | | | | | | | | | | | | | | | | | | | | | | | | | | |  |  |  |
| 4 | | | | | Ever heard of urinary schistosomiasis? | | | | | | | | Yes | | | | | | | |  | | | | | | | | No | | | |  | | | | | | | | | | | | | |  |  |  |
| If you answered “No” in the above question, skip this part and move on to PART 3. | | | | | | | | | | | | | | | | | | | | | | | | | | | | | | | | | | | | | | | | | | | | | | |  |  |  |
| 5 | | | | | If you answered “Yes”, where did you first hear it?  Only one answer can be selected | | | | | | | | | Health clinic/hospitals | | | | | | | | | | | | | | | | | | |  | | | | | | | | | | | | | |  |  |  |
|  |  |  |  |  |  |  |  |  |  |  |  |  |  | Mass media | | | | | | | | | | | | | | | | | | |  | | | | | | | | | | | | | |  |  |  |
|  |  |  |  |  |  |  |  |  |  |  |  |  |  | School/workshops | | | | | | | | | | | | | | | | | | |  | | | | | | | | | | | | | |  |  |  |
|  |  |  |  |  |  |  |  |  |  |  |  |  |  | Family/friends | | | | | | | | | | | | | | | | | | |  | | | | | | | | | | | | | |  |  |  |
|  |  |  |  |  |  |  |  |  |  |  |  |  |  | I do not remember | | | | | | | | | | | | | | | | | | |  | | | | | | | | | | | | | |  |  |  |
| **KNOWLEDGE OF SIGNS AND SYMPTOMS OF URINARY SCHISTOSOMIASIS** | | | | | | | | | | | | | | | | | | | | | | | | | | | | | | | | | | | | | | | | | | | | | | |  |  |  |
| In this section, choose yes or no against the items provided | | | | | | | | | | | | | | | | | | | | | | | | | | | | | | | | | | | | | | | | | | | | | | |  |  |  |
| The signs and symptoms of urinary schistosomiasis include; | | | | | | | | | | | | | | | | | | | | | | | | | | | | | | | | | | | | | | | | | | | | | | |  |  |  |
| 6 | | | | | Blood in urine | | | | | | | | | | | Yes | | | | | |  | | | | | | | No | | | |  | I do not know | | | | | | | | | |  | | | |  |  |
| 7 | | | | | Diarrhoea | | | | | | | | | | | Yes | | | | | |  | | | | | | | No | | | |  | I do not know | | | | | | | | | |  | | |  |  |  |
| 8 | | | | | Fever | | | | | | | | | | | Yes | | | | | |  | | | | | | | No | | | |  | I do not know | | | | | | | | | |  | | |  |  |  |
| 9 | | | | | Burning urination | | | | | | | | | | | Yes | | | | | |  | | | | | | | No | | | |  | I do not know | | | | | | | | | |  | | |  |  |  |
| 10 | | | | | Fatigue | | | | | | | | | | | | | | | Yes | |  | | | | | | | No | | | |  | I do not know | | | | | | | | | |  | | |  |  |  |
| 11 | | | | | Loss of appetite | | | | | | | | | | | | | | | Yes | |  | | | | | | | No | | | |  | I do not know | | | | | | | | | |  | | |  |  |  |
| 12 | | | | | Itching | | | | | | | | | | | | | | | Yes | |  | | | | | | | No | | | |  | I do not know | | | | | | | | | |  | | |  |  |  |
| 13 | | | | | Difficulty in walking | | | | | | | | | | | | | | | Yes | |  | | | | | | | No | | | |  | I do not know | | | | | | | | | |  | | |  |  |  |
| 14 | | | | | Inguinal hernia | | | | | | | | | | | | | | | Yes | |  | | | | | | | No | | | |  | I do not know | | | | | | | | | |  | | |  |  |  |
| 15 | | | | | I do not know | | | | | | | | | | | | | | | Yes | |  | | | | | | | No | | | |  | I do not know | | | | | | | | | |  | | |  |  |  |
| **KNOWLEDGE ON COMPLICATIONS OF URINARY SCHISTOSOMIASIS** | | | | | | | | | | | | | | | | | | | | | | | | | | | | | | | | | | | | | | | | | | | | | | |  |  |  |
| In this section, choose yes or no against the items provided | | | | | | | | | | | | | | | | | | | | | | | | | | | | | | | | | | | | | | | | | | | | | | |  |  |  |
| Late detection or untreated urinary schistosomiasis can lead to; | | | | | | | | | | | | | | | | | | | | | | | | | | | | | | | | | | | | | | | | | | | | | | |  |  |  |
| 16 | | | | | It stops children from growing well | | | | | | | | | | | | | Yes | | | | | | | | |  | | | | No | |  | I do not know | | | | | | | | | | | |  | | |  |
| 17 | | | | | Heart attack | | | | | | | | | | | | | Yes | | | | | | | | |  | | | | No | |  | I do not know | | | | | | | | | | | |  | | |  |
| 18 | | | | | HIV | | | | | | | | | | | | | Yes | | | | | | | | |  | | | | No | |  | I do not know | | | | | | | | | | | |  | | |  |
| 19 | | | | | It may lead to female genital schistosomiasis | | | | | | | | | | | | | Yes | | | | | | | | |  | | | | No | |  | I do not know | | | | | | | | | | | |  | | |  |
| 20 | | | | | I do not know | | | | | | | | | | | | | Yes | | | | | | | | |  | | | | No | |  | I do not know | | | | | | | | | | | |  | | |  |
| 21 | | | | | Bladder tumour | | | | | | | | | | | | | Yes | | | | | | | | |  | | | | No | |  | I do not know | | | | | | | | | | | |  | | |  |
| 22 | | | | | Liver failure | | | | | | | | | | | | | Yes | | | | | | | | |  | | | | No | |  | I do not know | | | | | | | | | | | |  | | |  |
| 23 | | | | | It stops children from learning well | | | | | | | | | | | | | Yes | | | | | | | | |  | | | | No | |  | I do not know | | | | | | | | | | | |  | | |  |
|  | | | | | | | | | | | | | | | | | | | | | | | | | | | | | | | | | | | | | | | | | | | | | | |  |  |  |
| **KNOWLEDGE ON MODE OF TRANSMISSION OF URINARY SCHISTOSOMIASIS** | | | | | | | | | | | | | | | | | | | | | | | | | | | | | | | | | | | | | | | | | | | | | | |  |  |  |
| In this section, answer by ticking the right answer | | | | | | | | | | | | | | | | | | | | | | | | | | | | | | | | | | | | | | | | | | | | | | |  |  |  |
| The mode of transmission of urinary schistosomiasis include; | | | | | | | | | | | | | | | | | | | | | | | | | | | | | | | | | | | | | | | | | | | | | | |  |  |  |
| 24 | | | Drinking contaminated water | | | | | | | | | | | | | | Yes | | | | | | | | |  | | | | No | | |  | I do not know | | | | | | |  | | | | | |  |  |  |
| 25 | | | Eating unwashed food | | | | | | | | | | | | | | Yes | | | | | | | | |  | | | | No | | |  | I do not know | | | | | | |  | | | | | |  |  |  |
| 26 | | | Walking barefoot in contaminated soil | | | | | | | | | | | | | | Yes | | | | | | | | |  | | | | No | | |  | I do not know | | | | | | |  | | | | | |  |  |  |
| 27 | | Mosquito bite | | | | | | | | | | | | | | | Yes | | | | | | | | |  | | | | No | | |  | I do not know | | | | | | |  | | | | | |  |  |  |
| 28 | Contact with contaminated water | | | | | | | | | | | | | | Yes | | | | | | | | | |  | | | | No | | | |  | I do not know | | | | | | |  | | | | | |  |  |  |
| 29 | I do not know | | | | | | | | | | | | | | Yes | | | | | | | | | |  | | | | No | | | |  | I do not know | | | | | | |  | | | | | |  |  |  |
| 30 | Insulting the gods | | | | | | | | | | | | | | Yes | | | | | | | | | |  | | | | No | | | |  | I do not know | | | | | | |  | | | | | |  |  |  |
| **KNOWLEDGE ON TREATMENT OF URINARY SCHISTOSOMIASIS** | | | | | | | | | | | | | | | | | | | | | | | | | | | | | | | | | | | | | | | | |  | | | | | |  |  |  |
| In this section, answer by ticking the right answer | | | | | | | | | | | | | | | | | | | | | | | | | | | | | | | | | | | | | | | | |  | | | | | |  |  |  |
| The treatment of urinary schistosomiasis includes; | | | | | | | | | | | | | | | | | | | | | | | | | | | | | | | | | | | | | | | | |  | | | | | |  |  |  |
| 31 | | | | Antibiotics | | | | | | | | | | | | | | | Yes | | | | | | | | |  | | | | No |  | I do not know | | | | | | | |  | | | | | | | |
| 32 | | | | Herbs | | | | | | | | | | | | | | | Yes | | | | | | | | |  | | | | No |  | I do not know | | | | | | | |  | | | | | | | |
| 33 | | | | Praziquantel | | | | | | | | | | | | | | | Yes | | | | | | | | |  | | | | No |  | I do not know | | | | | | | |  | | | | | | | |
| 34 | | | | I do not know | | | | | | | | | | | | | | | Yes | | | | | | | | |  | | | | No |  | I do not know | | | | | | | |  | | | | | | | |
| 35 | | | | It goes on its own | | | | | | | | | | | | | | | Yes | | | | | | | | |  | | | | No |  | I do not know | | | | | | | |  | | | | | | | |
| 36 | | | | Antivirals | | | | | | | | | | | | | | | Yes | | | | | | | | |  | | | | No |  | I do not know | | | | | | | |  | | | | |  |  |  |
| 37 | | | | Antifungals | | | | | | | | | | | | | | | Yes | | | | | | | | |  | | | | No |  | I do not know | | | | | | | |  | | | | |  |  |  |
| KNOWLEDGE ON PREVENTION OF URINARY SCHISTOSOMIASIS | | | | | | | | | | | | | | | | | | | | | | | | | | | | | | | | | | | | | | | | | | | | | | |  |  |  |
| Please tick the correct answer on how to prevent urinary schistosomiasis. | | | | | | | | | | | | | | | | | | | | | | | | | | | | | | | | | | | | | | | | | | | | | | |  |  |  |
| 38 | | | | Wearing condom before sexual intercourse | | | | | | | | | | | | | | | Yes | | | | | | | | |  | | | | No |  | I do not know | | | | | | | | |  | | | | |  |  |
| 39 | | | | Avoid urinating into water bodies | | | | | | | | | | | | | | | Yes | | | | | | | | |  | | | | No |  | I do not know | | | | | | | | |  | | | |  |  |  |
| 40 | | | | Avoid swimming or playing in contaminated water | | | | | | | | | | | | | | | Yes | | | | | | | | |  | | | | No |  | I do not know | | | | | | | | |  | | | |  |  |  |
| 41 | | | | Boil water from contaminated source before using | | | | | | | | | | | | | | | Yes | | | | | | | | |  | | | | No |  | I do not know | | | | | | | | |  | | | |  |  |  |
| 42 | | | | Avoid defecating into water bodies | | | | | | | | | | | | | | | Yes | | | | | | | | |  | | | | No |  | I do not know | | | | | | | | |  | | | |  |  |  |
| 43 | | | | Sleeping under insecticide treated mosquito net | | | | | | | | | | | | | | | Yes | | | | | | | | |  | | | | No |  | I do not know | | | | | | | | |  | | | |  |  |  |
| 44 | | | | Vaccination | | | | | | | | | | | | | | | Yes | | | | | | | | |  | | | | No |  | I do not know | | | | | | | | | | |  | | |  |  |
| 45 | | | | Using insecticides | | | | | | | | | | | | | | | Yes | | | | | | | | |  | | | | No |  | I do not know | | | | | | | | | | |  | | |  |  |
| 46 | | | | Mass treatment of communities with praziquantel | | | | | | | | | | | | | | | Yes | | | | | | | | |  | | | | No |  | I do not know | | | | | | | | | | |  | | |  |  |
| PART 3 | | | | | | | | | | | | | | | | | | | | | | | | | | | | | | | | | | | | | | | | | | | | | | |  |  |  |
| **AWARENESS OF FGS** | | | | | | | | | | | | | | | | | | | | | | | | | | | | | | | | | | | | | | | | | | | | | | |  |  |  |
| In this section, answer by ticking the right answer | | | | | | | | | | | | | | | | | | | | | | | | | | | | | | | | | | | | | | | | | | | | | | |  |  |  |
| 47 | | | | | | | | | Ever heard of female genital schistosomiasis? | | | | | | | | | | Yes | | | | |  | | | | | No | | | |  | | | | | | | | | | | | | |  |  |  |
| If you answered “No” in the above question, do not continue with the subsequent questions in this part. | | | | | | | | | | | | | | | | | | | | | | | | | | | | | | | | | | | | | | | | | | | | | | |  |  |  |
| 48 | | | | | | | If you answered “Yes”, where did you first hear it?  Only one answer can be selected. | | | | | Health clinic/hospitals | | | | | | | | | | | | | | | | | | | | |  | | | | | | | | | | | | | |  |  |  |
|  |  |  |  |  |  |  |  |  |  |  |  | School / Workshops | | | | | | | | | | | | | | | | | | | | |  | | | | | | | | | | | | | |  |  |  |
|  |  |  |  |  |  |  |  |  |  |  |  | Mass media | | | | | | | | | | | | | | | | | | | | |  | | | | | | | | | | | | | |  |  |  |
|  |  |  |  |  |  |  |  |  |  |  |  | Family/friends | | | | | | | | | | | | | | | | | | | | |  | | | | | | | | | | | | | |  |  |  |
|  |  |  |  |  |  |  |  |  |  |  |  | I do not remember | | | | | | | | | | | | | | | | | | | | |  | | | | | | | | | | | | | |  |  |  |
| **KNOWLEDGE ON SIGNS AND SYMPTOMS OF FEMALE GENITAL SCHISTOSOMIASIS** | | | | | | | | | | | | | | | | | | | | | | | | | | | | | | | | | | | | | | | | | | | | | | |  |  |  |
| In this section, choose yes or no against the items provided | | | | | | | | | | | | | | | | | | | | | | | | | | | | | | | | | | | | | | | | | | | | | | |  |  |  |
| The signs and symptoms of female genital schistosomiasis include; | | | | | | | | | | | | | | | | | | | | | | | | | | | | | | | | | | | | | | | | | | | | | | |  |  |  |
| 49 | | | | | | | | Vaginal discharge | | | | | | | | | | | Yes | | | | |  | | | | | No | | | |  | | | | I do not know | | | | | | | | |  |  |  |  |
| 50 | | | | | | | | Blood discharge | | | | | | | | | | | Yes | | | | |  | | | | | No | | | |  | | | | I do not know | | | | | | | | |  |  |  |  |
| 51 | | | | | | | | Bleeding after intercourse or spotting | | | | | | | | | | | Yes | | | | |  | | | | | No | | | |  | | | | I do not know | | | | | | | | |  |  |  |  |
| 52 | | | | | | | | Genital itching or burning sensation | | | | | | | | | | | Yes | | | | |  | | | | | No | | | |  | | | | I do not know | | | | | | | | |  |  |  |  |
| 53 | | | | | | | | Pelvic pain or pain during or after intercourse | | | | | | | | | | | Yes | | | | |  | | | | | No | | | |  | | | | I do not know | | | | | | | | |  |  |  |  |
| 54 | | | | | | | | I do not know | | | | | | | | | | | Yes | | | | |  | | | | | No | | | |  | | | | I do not know | | | | | | | | |  |  |  |  |
| **KNOWLEDGE ON COMPLICATIONS OF FGS** | | | | | | | | | | | | | | | | | | | | | | | | | | | | | | | | | | | | | | | | | | | | | | |  |  |  |
| In this section, choose yes or no against the items provided | | | | | | | | | | | | | | | | | | | | | | | | | | | | | | | | | | | | | | | | | | | | | | |  |  |  |
| Late detection or untreated female genital schistosomiasis can lead to; | | | | | | | | | | | | | | | | | | | | | | | | | | | | | | | | | | | | | | | | | | | | | | |  |  |  |
| 55 | | | | | | | | Fibroids | | | | | | | | | | | Yes | | | | |  | | | | | No | | | |  | | I do not know | | |  | | | | | | | | |  |  |  |
| 56 | | | | | | | | Cervical Cancer | | | | | | | | | | | Yes | | | | |  | | | | | No | | | |  | | I do not know | | |  | | | | | | | | |  |  |  |
| 57 | | | | | | | | Gonorrhoea | | | | | | | | | | | Yes | | | | |  | | | | | No | | | |  | | I do not know | | |  | | | | | | | | |  |  |  |
| 58 | | | | | | | | Syphilis | | | | | | | | | | | Yes | | | | |  | | | | | No | | | |  | | I do not know | | |  | | | | | | | | |  |  |  |
| 59 | | | | | | | | Candidiasis | | | | | | | | | | | Yes | | | | |  | | | | | No | | | |  | | I do not know | | |  | | | | | | | | |  |  |  |
| 60 | | | | | | | | Ectopic Pregnancy | | | | | | | | | | | Yes | | | | |  | | | | | No | | | |  | | I do not know | | |  | | | | | | | | |  |  |  |
| 61 | | | | | | | | Infertility | | | | | | | | | | | Yes | | | | |  | | | | | No | | | |  | | I do not know | | |  | | | | | | | | |  |  |  |
| 62 | | | | | | | | I do not Know | | | | | | | | | | | Yes | | | | |  | | | | | No | | | |  | | I do not know | | |  | | | | | | | | |  |  |  |
| 63 | | | | | | | | Abortion | | | | | | | | | | | Yes | | | | |  | | | | | No | | | |  | | I do not know | | |  | | | | | | | | |  |  |  |
| 64 | | | | | | | | Genital ulcers | | | | | | | | | | | Yes | | | | |  | | | | | No | | | |  | | I do not know | | |  | | | | | | | | |  |  |  |
| **KNOWLEDGE ON TRANSMISSION OF FEMALE GENITAL SCHISTOSOMIASIS** | | | | | | | | | | | | | | | | | | | | | | | | | | | | | | | | | | | | | | | | | | | | | | |  |  |  |
| In this section, choose yes or no against the items provided | | | | | | | | | | | | | | | | | | | | | | | | | | | | | | | | | | | | | | | | | | | | | | |  |  |  |
| The transmission of female genital schistosomiasis include; | | | | | | | | | | | | | | | | | | | | | | | | | | | | | | | | | | | | | | | | | | | | | | |  |  |  |
| 65 | | | | | | | | Playing with soil | | | | | | | | | | | Yes | | | | |  | | | | | No | | | |  | | | I do not know | | | |  | | | | | | |  |  |  |
| 66 | | | | | | | | Eating contaminated food | | | | | | | | | | | Yes | | | | |  | | | | | No | | | |  | | | I do not know | | | |  | | | | | | |  |  |  |
| 67 | | | | | | | | Drinking untreated water | | | | | | | | | | | Yes | | | | |  | | | | | No | | | |  | | | I do not know | | | |  | | | | | | |  |  |  |
| 68 | | | | | | | | Unprotected sexual intercourse | | | | | | | | | | | Yes | | | | |  | | | | | No | | | |  | | | I do not know | | | |  | | | | | | |  |  |  |
| 69 | | | | | | | | Swimming/bathing in infested water | | | | | | | | | | | Yes | | | | |  | | | | | No | | | |  | | | I do not know | | | |  | | | | | | |  |  |  |
| 70 | | | | | | | | Dirty hands | | | | | | | | | | | Yes | | | | |  | | | | | No | | | |  | | | I do not know | | | |  | | | | | | |  |  |  |
| 71 | | | | | | | | I do not know | | | | | | | | | | | Yes | | | | |  | | | | | No | | | |  | | | I do not know | | | |  | | | | | | |  |  |  |
| 72 | | | | | | | | Physical contact with an infected person | | | | | | | | | | | Yes | | | | |  | | | | | No | | | |  | | | I do not know | | | |  | | | | | | |  |  |  |
| 73 | | | | | | | | Improper use of family planning method | | | | | | | | | | | Yes | | | | |  | | | | | No | | | |  | | | I do not know | | | |  | | | | | | |  |  |  |
| 74 | | | | | | | | Punishment by the gods | | | | | | | | | | | Yes | | | | |  | | | | | No | | | |  | I do not know | | | | | | | |  | | | | |  |  |  |
| **KNOWLEDGE ON TREATMENT OF FEMALE GENITAL SCHISTOSOMIASIS** | | | | | | | | | | | | | | | | | | | | | | | | | | | | | | | | | | | | | | | | | | | | | | |  |  |  |
| In this section, choose yes or no against the items provided | | | | | | | | | | | | | | | | | | | | | | | | | | | | | | | | | | | | | | | | | | | | | | |  |  |  |
| The treatment of female genital schistosomiasis includes; | | | | | | | | | | | | | | | | | | | | | | | | | | | | | | | | | | | | | | | | | | | | | | |  |  |  |
| 75 | | | | | | | | Antibiotics | | | | | | | | | | | Yes | | | | |  | | | | | No | | | |  | I do not know | | | | |  | | | | | | | |  |  |  |
| 76 | | | | | | | | Praziquantel | | | | | | | | | | | Yes | | | | |  | | | | | No | | | |  | I do not know | | | | |  | | | | | | | |  |  |  |
| 77 | | | | | | | | Herbal medications | | | | | | | | | | | Yes | | | | |  | | | | | No | | | |  | I do not know | | | | |  | | | | | | | |  |  |  |
| 78 | | | | | | | | Antivirals | | | | | | | | | | | Yes | | | | |  | | | | | No | | | |  | I do not know | | | | |  | | | | | | | |  |  |  |
| 79 | | | | | | | | Contraception | | | | | | | | | | | Yes | | | | |  | | | | | No | | | |  | I do not know | | | | |  | | | | | | | |  |  |  |
| 80 | | | | | | | | Antifungals | | | | | | | | | | | Yes | | | | |  | | | | | No | | | |  | I do not know | | | | |  | | | | | | | |  |  |  |
| 81 | | | | | | | | I do not know | | | | | | | | | | | Yes | | | | |  | | | | | No | | | |  | I do not know | | | | |  | | | | | | | |  |  |  |
| KNOWLEDGE ON PREVENTION OF FEMALE GENITAL SCHISTOSOMIASIS | | | | | | | | | | | | | | | | | | | | | | | | | | | | | | | | | | | | | | | | | | | | | | |  |  |  |
| Please tick the correct answer on how to prevent female genital schistosomiasis. | | | | | | | | | | | | | | | | | | | | | | | | | | | | | | | | | | | | | | | | | | | | | | |  |  |  |
| 82 | | | | | | Wearing condom before sexual intercourse | | | | | | | | | | | | | Yes | | | |  | | | | | | No | | | |  | I do not know | | | | | | | |  | | | | | |  |  |
| 83 | | | | | | Avoid urinating into water bodies | | | | | | | | | | | | | Yes | | | |  | | | | | | No | | | |  | I do not know | | | | | | | |  | | | | |  |  |  |
| 84 | | | | | | Avoid swimming or playing in contaminated water | | | | | | | | | | | | | Yes | | | |  | | | | | | No | | | |  | I do not know | | | | | | | |  | | | | |  |  |  |
| 85 | | | | | | Avoid defecating into water bodies | | | | | | | | | | | | | Yes | | | |  | | | | | | No | | | |  | I do not know | | | | | | | |  | | | | |  |  |  |
| 86 | | | | | | Boil water from contaminated source before using | | | | | | | | | | | | | Yes | | | |  | | | | | | No | | | |  | I do not know | | | | | | | |  | | | | |  |  |  |
| 87 | | | | | | Sleeping under insecticide treated mosquito net | | | | | | | | | | | | | Yes | | | |  | | | | | | No | | | |  | I do not know | | | | | | | |  | | | | |  |  |  |
| 88 | | | | | | Vaccination | | | | | | | | | | | | | Yes | | | |  | | | | | | No | | | |  | I do not know | | | | | | | |  | | | | |  |  |  |
| 89 | | | | | | Using insecticides | | | | | | | | | | | | | Yes | | | |  | | | | | | No | | | |  | I do not know | | | | | | | |  | | | | |  |  |  |
| 90 | | | | | | Mass treatment of communities with praziquantel | | | | | | | | | | | | | Yes | | | |  | | | | | | No | | | |  | I do not know | | | | | | | |  | | | | |  |  |  |
