# Supplementary material for: Knowledge of female genital schistosomiasis and urinary schistosomiasis among final-year midwifery students in the Volta Region of Ghana
Source: PLoS One. 2024 May 2;19(5):e0302554. doi: 10.1371/journal.pone.0302554 (PMC11065279; doi:10.1371/journal.pone.0302554)
Supplement: Attachment — Submitted filename: RESPONSE TO REVIEWERS new.docx [file pone.0302554.s006.docx]

**RESPONSES TO REVIEWERS’ COMMENTS AND JOURNAL REQUIREMENTS**

**JOURNAL REQUIREMENTS**

1. Copyright issues with Figure 1.

**Response:** *On the matter of the potential copyrights relating to Figure 1, the authors have chosen to remove the said image/figure. We have no means of identifying the original copyright holders and therefore will not take this path. Hence, Figure 1 is removed from the submission and all references to it in the manuscript have also been taken out.*

**RESPONSES TO COMMENTS FROM REVIEWER #1**

1. Remove unnecessary capitalization of first letters and abbreviations throughout the write up.

**Response:** *All unnecessary capitalization of first letters and abbreviations have been removed as suggested. Only those deemed necessary have been left.*

2. The title should reflect what is contained in the paper. While the title is about knowledge of urinary schistosomiasis and female genital schistosomiasis, in the main body, authors are mainly featuring FGS at expense of urinary schistosomiasis also appearing in the title. It is either the title to be rephrased or address the imbalance in the main body.

**Response:** *This has been addressed. We have reported on knowledge levels regarding urinary schistosomiasis as well and provided appropriate citations. Please see the second paragraph of the ‘Introduction’ which reads as follows;*

3. The title of the paper please write female genital schistosomiasis in full. Since FGS is a subset of urinary schistosomiasis, authors should consider interposing these in the title to start with the broad urinary schistosomiasis then specifically with FGS.

**Response:** *Female genital schistosomiasis has been written out fully and the title maintained.*

4. As done with Background, Methodology, Findings and Conclusions should also be presented separately in the abstract.

**Response:** *This has been corrected and we are grateful to the reviewer for drawing our attention to this anomaly. This came about because the manuscript was originally formatted for and submitted to a sister PLOS journal (PLOS NTDs). They advised it was more suitable for PLOS ONE and subsequently forwarded it.*

5. Since the paper is assessing knowledge of urinary schistosomiasis and FGS, authors should first separately introduce the context of urinary schistosomiasis before delving into FGS.

**Response:** *We agree with the reviewer on this and have presented a slight variation in the revised manuscript. In the opening paragraph of the ‘Introduction’, we have established the link between urinary schistosomiasis (US) and female genital schistosomiasis (FGS). We chose to describe the clinical implications of FGS first to emphasize our focus on it. Subsequently, we highlighted knowledge levels/ local prevalence of US and compared it to that of FGS. Eventually, we went into the problem statement, study objective and study significance where it has been emphasized that we added on US for context and to enable comparison of knowledge base of these two conditions. Thank you.*

6. Lines 108-114 about urinary schistosomiasis should come earlier before FGS as pointed out in above comment.

**Response:** *This has been done (though with a slightly varying approach).*

7. Lines 115-135 authors should be justifying why it was necessary to carry out this study which is about the need for health workers (generally - not just midwives) having adequate knowledge about urinary schistosomiasis and FGS. Remove the ‘final year midwifery students’ bit from this part of the Introduction to Methods where study population is described.

**Response:** *The last sentence in the last-but-two paragraph of the ‘Introduction’ describes fall- outs from a lack of adequate knowledge of FGS among health care workers and justifies the need to assess this knowledge among health care workers including those still in training (as outlined in the new opening sentence of the penultimate paragraph*……” It is therefore important to assess the knowledge of health care providers, including those still in training, regarding FGS.”).

*The authors humbly disagree with the reviewer concerning moving ‘the final year midwifery students’ bit from the Introduction to the Methods section. The authors are of the opinion that the specific study population and the justification for that study population need to be clarified early in the context of defining the problem statement and the study significance.*

8. Study design, study site description and study population: Authors tend to merge several sub-sections into one making it difficult for readers to follow the write up. Consider breaking down the sub-heading into separate sub-sections i.e. Study design; Study area; Study population to make it easier for readers to follow the presentation.

**Response:** *As suggested and to improve clarity, ‘Study design and population’ has now been made a separate sub-section while ‘Study site description’ is another sub-section. Please see the version with track changes and the clean version as well.*

9. In presentation of the results, authors should start by presenting the results and refer to a Table or Figure towards the end not at the beginning as they have done with Table 1 in line 224.

**Response:** *The recommendation has been accepted and revision done accordingly. Presentation of participants’ background characteristics (which have been so defined to include whether they have ever heard of urinary schistosomiasis and female genital schistosomiasis) now reads as follows;*

“All planned 193 final-year students took part in the survey. Hohoe and Keta NMTC had similar numbers of participants in the study; 71 (36.8%) and 73 (37.8%). Close to half (48.7%) were in the age group 23-27 years while 31 (16.1%) were in the youngest age group, 18-22 years One hundred and twenty-three (64.1%) participants indicated they had heard about urinary schistosomiasis. Comparatively, just about a quarter (23.3%) reported ever hearing of female genital schistosomiasis (see Table 1).”

10. Start by presenting the total number of participants before breaking down according to sites, age groups etc. i.e. “The study enrolled the total of XX participant of which ZZ (%) were from site A, YY (%) site B….”

**Response:** *The recommendation is accepted and revision has been done accordingly. The relevant section now reads as follows;*

“All planned 193 final-year students took part in the survey. Hohoe and Keta NMTC had similar numbers of participants in the study; 71 (36.8%) and 73 (37.8%). Close to half (48.7%) were in the age group 23-27 years while 31 (16.1%) were in the youngest age group, 18-22 years One hundred and twenty-three (64.1%) participants indicated they had heard about urinary schistosomiasis. Comparatively, just about a quarter (23.3%) reported ever hearing of female genital schistosomiasis (see Table 1).”

11. Present numbers in digits not in words as done in line 227.

**Response:** *We thank the reviewer for his comments. In the portion under reference, the number begins a sentence. The convention is to have it in words under such circumstances. The authors therefore prefer to maintain the status quo.*

12. Ensure that correct results are presented under a correct heading. The first sub-heading is about “Background characteristics of participants” where authors should present characteristics of the participants such as age, sex, school etc. with help of a revised Table 1 (without the last two variables which need to be reported under own sub-headings). In line with the title of this paper authors should report for both urinary schistosomiasis and FGS.

**Response:** *We are grateful to the reviewer for his comments. In the particular case of Table 1, the last two variables “ever heard of urinary schistosomiasis” and “ever heard of female genital schistosomiasis” have been defined to be part of ‘Background Characteristics’ (which is broader than just demographic characteristics) in the Methods section. The authors opine that ‘ever heard’ does not exactly equate to ‘knowledge’ and thus the two should not be together.*

*Under Methods, the Study procedure and data collection sub-section has been revised to read;*

“……….The questionnaire was divided into three main parts. The first part collected data on the background characteristics of participants which included age, institution attended and whether they had ever heard of urinary and female genital schistosomiasis……….”

On account of this, the authors prefer to maintain Table 1 in its current form.

13. The next sub-heading is about “Knowledge of Female Genital Schistosomiasis” should come earlier where all results of participants’ knowledge about FGS (including one (last) variable in Table 1 should be presented. Similarly, authors should consider preceding this section with a sub-heading on “Knowledge of Urinary Schistosomiasis” on line 277 to appear earlier before FGS as indicated in 2 and 4 above. Tables 2-5 and their respective narratives should be realigned with appropriate sub-heading.

**Response:** *This is acknowledged. The suggestions have been adopted and presented in a slightly different way to improve clarity. A new sub-section “Participants’ source of information about urinary and female genital schistosomiasis” has been introduced and the old Table 2 is now Table 3. “Knowledge of urinary schistosomiasis” now precedes that of female genital schistosomiasis and the tables / table numbers have been realigned with their sub-headings appropriately.*

14. The discussion should be re-organized to include findings for both urinary schistosomiasis and FGS (making sure that urinary schistosomiasis coming before FGS).

**Response:** *We have included findings on urinary schistosomiasis in the opening paragraph of the Discussion. It now reads as follows;*

“………Among those who reported hearing about urinary schistosomiasis before, about 93% correctly chose ‘blood in urine’ as a symptom while 68% wrongly selected inguinal hernia as a symptom or indicated they did not know if it was or not. Nearly 70% of these specific participants indicated that urinary schistosomiasis was transmitted by drinking contaminated water.”

*Findings on both urinary schistosomiasis and female genital schistosomiasis have been discussed vis-à-vis each other to better relate comparisons in practically all of the paragraphs of the ‘Discussion’.*

**RESPONSES TO COMMENTS FROM REVIEWER #2**

1. The paper is of interest to all health care practitioners more specifically those working in areas of sexual and reproductive health, and I believe in all parts of SSA where schistosomiasis is endemic. Lines 55-64 speak to the primary purpose which was to assess final year midwifery students in the Volta Region of Ghana an area endemic for urinary schistosomiasis for knowledge of causes, transmission, presentation, treatment and prevention of FSG. Authors highlight key issues: missed diagnosis due to limited knowledge etc. During training, particularly in the clinical area aren't women screened schistosomiasis for family planning, particularly for IUCD and for prenatal care to rule out both STI and schistosomiasis given that it is endemic in that region; when the activities on Neglected Tropical Diseases is aiming for a global elimination?

**Response:** *The authors are grateful to the reviewer for the kind comments.*

*We do screen for STIs as part of work-ups for uptake of IUCD and for prenatal care as mentioned. Unfortunately, schistosomiasis has never been on the agenda in this respect. As mentioned in the ‘Background’ of the manuscript, some vaginal discharges may be caused by FGS but because our minds were not focused to it, the tendency has been to treat with antibiotics. In some circumstances, one may see some improvement but this is soon followed by a recurrence. In others, there is no improvement at all since not all vaginal discharge is caused by bacteria and patients keep on moving from one health facility to the next seeking for solutions.*

*In summary, FGS has not been the radar at all. In the routine clinical care and family planning services in Ghana, women are not routinely screened for schistosomiasis or FGS unless they are symptomatic. In many endemic areas like Ghana, there is rather MDA from time to time to help control/prevent urinary schistosomiasis/FGS. Prenatal care includes routine urine examination which can pick up schistosomiasis but not FGS. Besides, we don’t do routine colposcopy for prenatal services. Even in performing colposcopy for other reasons, most health care providers lack the awareness and/or knowledge to pick up FGS*

2. while I agree with recommendations made, one cannot help but wonder what the Ghana national policy on prevention and control says about FSG? No reference was made to. Shouldn't all health training institutions curricula for doctors, nurses, lab technicians/scientists for example include schistosomiasis? given the limited knowledge regarding causes, diagnostic, treatment and prevention and control?

**Response:** *Ghana has a programme for NTD control that oversees schistosomiasis, lymphatic filariasis, onchocerciasis, yaws and others. However, FGS has never been in the picture. Schistosomiasis, as a topic, is in the curriculum of the health training institutions but there is really no mention of FGS. Thus, there has never been any established education on FGS. Until recent awareness creation by the FGS FAST package by a partnership of global and local actors to fund and support awareness creation about FGS, most of the faculty in health training institutions (including very senior gynaecologists as we realized in a recent West African conference in Lome, Togo) were not aware of FGS. It is expected that the study findings will contribute to making a case for incorporating FGS into the curriculum of health training institutions.*
