# Supplementary material for: Knowledge of female genital schistosomiasis and urinary schistosomiasis among final-year midwifery students in the Volta Region of Ghana
Source: PLoS One. 2024 May 2;19(5):e0302554. doi: 10.1371/journal.pone.0302554 (PMC11065279; doi:10.1371/journal.pone.0302554)
Supplement: Attachment — Submitted filename: RESPONSE TO REVIEWERS 24032024.docx [file pone.0302554.s008.docx]

**RESPONSES TO SECOND ROUND OF COMMENTS FROM REVIEWER #1**

1) Lines 110-119 are repeating what has already been reported in lines 87-95 citing the same Nigerian study [13].

**Response:** The authors are thankful to the reviewer for this comment. This specific comment is from the last review and was addressed. Thus, lines 110-119 are not a repetition of lines 87-95. The Nigerian study [13] has been used in both portions to represent differing contexts.

2) Line 126-127 – The sentence “It is therefore important to assess the knowledge of health care providers, including those still in training, regarding FGS.” should be moved to the next paragraph at the beginning of line 138.

**Response:** This has been done as suggested.

3) Lines 139-141 – authors write “The Volta region was chosen because, historically, communities along the Volta Lake have been known to be endemic for urinary schistosomiasis [15, 27].” Since the purpose of this study was to assess the acquired knowledge/training about US and FGS among midwifery students it is not relevant whether the chosen area was to be an endemic for schistosomiasis or not.

**Response:** We thank the reviewer for his insightful comments. In this particular instance, we reckoned that since US is endemic in the communities referred to, the trainee midwives would, at least, have a minimum acceptable level of knowledge of US and FGS. The trainee midwives are, at the barest minimum, part of the population in these areas and this has been alluded to in the Discussion section originally. The section in question has been revised to include a second reason for working in Volta Region and now reads as;

“The Volta region was chosen because communities along the Volta Lake have been known to be endemic for urinary schistosomiasis [15, 27] and also for ease of accessibility to the students as all but one of the investigators are based in the region.”

4) [A previous comment]: Authors tend to merge several sub-sections into one making it difficult for readers to follow the write up. Consider breaking down the sub-heading into separate sub-sections i.e. Study design; Study area; Study population to make it easier for readers to follow the presentation. This comment remains unresolved. For example, in the revised manuscript, authors have chosen to merge ‘study design with population’ yet the content therein have elements of time and place of the study not related to the subheading. There are so many instances of repetitions within the subheadings, the authors are therefore invited to critically re-examine each sub-heading and the contents to ensure that they are not repeated, easy and logical for a reader to follow.

**Response:** This has been done as suggested. The ‘Study population’ now stands alone just before sample size estimation. The period of data collection has been moved to ‘Study procedures and data collection’. The authors acknowledge and appreciate the differences in presentation style but we would like to maintain the hint of ‘place of study’ under the ‘Study design’.

5) Study site description – authors are presenting data from OPD or DHMIS2 probably to justify why the areas were selected for the study. This again raises the question in 3 above, what have these statistics to do with choice of the midwifery students who were the study population as opposed to community members whose data was recorded at OPD/DHMIS2?

**Response:** The authors are grateful to the reviewer for the opportunity to explain our rationale. First, we did not exactly ‘select’ the study areas. They are included by default because those areas are where the three midwifery training institutions in the Volta Region are located. This was indicated earlier in the Methods section. The health facility data provided give context of the burden of urinary schistosomiasis (US) in the various areas as part of the study site description. To our knowledge and as much as possible, study site description ought to be done with a focus on some aspects of the subject matter in question. Since ours is the first evaluation among midwifery students in our country, we reckoned we could at least minimally describe the amount of documented cases of US. The said data did not play any role at all in the choice of the study population.

6) Sample size estimation should be preceded by a ‘study population description’ sub-section, either separately or merged

**Response:** This has been done. ‘Study population’ stands alone just before the sub-section on sample size estimation.

7) Study procedures and data collection – line 184 authors write “All final-year midwifery students in the Volta region were included in the study.” This sentence is contradictory with what is immediately written up to line 197.

**Response:** We thank the reviewer for picking up this oversight. The sentence in question is now revised to read as;

“All final-year midwifery students in the Volta region were eligible for inclusion in the study.”

8) Since the authors indicated that in Ghana only US unlike FGS is part of curricula in medical training institutions. The authors need to explore and discuss the obtained result in the present study that indicated schools/workshops as the main source of information about US (54.9%) and FGS (59%) for many of the midwifery students that were interviewed. Were these schools/workshops where they got information at the same training colleges they were enrolled at the time of the study or different ones since these are not yet qualified midwives?

**Response:** This study finding was originally discussed over lines 336-349. We have further revised and strengthened the Discussion on these observations. Specifically, lines 343-352 now read as;

“……In comparison, FGS is so far not part of the curriculum in their training. It is probable then that the knowledge of FGS was more from extracurricular workshops organized for these midwives. For instance, it is possible that their tutors may have been involved in training workshops about schistosomiasis and FGS organized by the Volta River Authority (VRA) and may have casually handed down such information during lectures on related topics such as anaemia in pregnancy. In addition to the Neglected Tropical Diseases Control Programme in Ghana, the VRA is also involved in schistosomiasis control along the Volta Lake. Nevertheless, here in Ghana, the named sources may have to be taken with caution since FGS is still a relatively new ‘discovery’ that is now being brought to the attention of even clinicians.”

9) In lines 347-350 authors write “Participants from Keta NMTC had the highest proportion of participants who had heard about urinary schistosomiasis followed by UHAS and Hohoe NMTC respectively. This could be because Keta has a lot more water bodies than the other places and possibly a higher burden of urinary schistosomiasis.” Can authors explain the connection between the environmental setting of the area with the respondent midwifery students’ acquired knowledge/training in relation to comments 3 and 5 above?

**Response:** In relation to comment 5 specifically, we sought to describe the study area with respect to documented occurrences of US cases. The argument (if the authors may be allowed to call it so) with respect to comments 3 and 9 has been that the midwifery students, being part of the general population and also being part of the hospital setting at different times in their training, may be presumed have some minimum level of awareness / knowledge of US in areas with relatively more appreciable burdens of US. The particular example of Keta is made where the students live within communities rather than secluded hostels and may be more aware of mass drug administration efforts for US.

The section (lines 358-369) in question is now revised to read as follows;

“Keta NMTC had the highest proportion of participants who had heard about urinary schistosomiasis followed by UHAS and Hohoe NMTC respectively. This could be because Keta has a lot more water bodies than the other places and possibly a higher burden of urinary schistosomiasis which may underpin relatively more awareness among these students as their training also involves the hospital and community setting. The mass drug administration for prevention of Schistosoma infection in that area may also have created more awareness. Also in Keta NMTC, most of the students live within the community rather than in secluded hostels and so may be more aware of or involved in community activities pertaining to urinary schistosomiasis than in UHAS and Hohoe where the students live in hostels on the schools’ premises. This relation between the school attended and whether the respondents had ever heard of urinary schistosomiasis was not observed in the case of FGS. This may arise from the general deficiency in knowledge of FGS.”

10) Another limitation of the study is that it was conducted among pre-service students, hindering its generalizability to the in-service group of health worker cadres who acquire more exposure and experience while interacting with community members than the students who are mostly attending lessons in their colleges

**Response:** The suggested limitation has been included.

11) The first sentence should qualify “[…] among final year midwifery students in the Volta Region, Ghana”.

**Response:** This was an oversight and we thank the reviewer for detecting it. The suggested qualification has been inserted appropriately and the sentence now reads as;

“This study assessed the awareness of US and FGS among final year midwifery students in the Volta region of Ghana as well as the knowledge of their signs, symptoms, complications, treatment and prevention.”
