# Supplementary material for: Knowledge of female genital schistosomiasis and urinary schistosomiasis among final-year midwifery students in the Volta Region of Ghana
Source: PLoS One. 2024 May 2;19(5):e0302554. doi: 10.1371/journal.pone.0302554 (PMC11065279; doi:10.1371/journal.pone.0302554)
Supplement: Attachment — Submitted filename: Reviewer comments.pdf [file pone.0302554.s005.pdf]

Knowledge of Female Genital and Urinary Schistosomiasis among final-year midwifery students in the Volta Region of Ghana.

Thank you for the opportunity to review this paper. This is a paper describing an assessment of knowledge level for urinary schistosomiasis and female genital schistosomiasis among final year midwifery students in Ghana. The authors should be commended for writing this paper on an important topic of neglected tropical diseases. However, there are some areas that will need to be revised to improve general quality of the paper. Below are general and specific comments for the authors' consideration:

A. General comments

1) Remove unnecessary capitalization of first letters and abbreviations throughout the write up.
2) The title should reflect what is contained in the paper. While the title is about knowledge of urinary schistosomiasis and female genital schistosomiasis, in the main body, authors are mainly featuring FGS at expense of urinary schistosomiasis also appearing in the title. It is either the title to be rephrased or address the imbalance in the main body.

B. Title and abstract

2) The title of the paper please write female genital schistosomiasis in full. Since FGS is a subset of urinary schistosomiasis, authors should consider interposing these in the title to start with the broad urinary schistosomiasis then specifically with FGS.
3) As done with Background, Methodology, Findings and Conclusions should also be presented separately in the abstract.

C. Introduction

4) Since the paper is assessing knowledge of urinary schistosomiasis and FGS, authors should first separately introduce the context of urinary schistosomiasis before delving into FGS.
5) Lines 108-114 about urinary schistosomiasis should come earlier before FGS as pointed out in above comment.
6) Lines 115-135 authors should be justifying why it was necessary to carry out this study which is about the need for health workers (generally - not just midwives) having adequate knowledge about urinary schistosomiasis and FGS. Remove the 'final year midwifery students' bit from this part of the Introduction to Methods where study population is described.

D. Methodology

7) Study design, study site description and study population: Authors tend to merge several sub-sections into one making it difficult for readers to follow the write up. Consider breaking down the sub-heading into separate sub-sections i.e. Study design; Study area; Study population to make it easier for readers to follow the presentation.

E. Results

8) In presentation of the results, authors should start by presenting the results and refer to a Table or Figure towards the end not at the beginning as they have done with Table 1 in line 224.

9) Start by presenting the total number of participants before breaking down according to sites, age groups etc. i.e. "The study enrolled the total of XX participant of which ZZ (%) were from site A, YY (%) site B…."

10) Present numbers in digits not in words as done in line 227.

11) Ensure that correct results are presented under a correct heading. The first sub-heading is about "Background characteristics of participants" where authors should present characteristics of the participants such as age, sex, school etc. with help of a revised Table 1 (without the last two variables which need to be reported under own sub-headings). In line with the title of this paper authors should report for both urinary schistosomiasis and FGS. The next sub-heading is about "Knowledge of Female Genital Schistosomiasis" should come earlier where all results of participants' knowledge about FGS (including one (last) variable in Table 1 should be presented. Similarly, authors should consider preceding this section with a sub-heading on "Knowledge of Urinary Schistosomiasis" on line 277 to appear earlier before FGS as indicated in 2 and 4 above. Tables 2-5 and their respective narratives should be realigned with appropriate sub-heading.

F. Discussions

12) The discussion should be re-organized to include findings for both urinary schistosomiasis and FGS (making sure that urinary schistosomiasis coming before FGS.
