# Supplementary material for: Knowledge of female genital schistosomiasis and urinary schistosomiasis among final-year midwifery students in the Volta Region of Ghana
Source: PLoS One. 2024 May 2;19(5):e0302554. doi: 10.1371/journal.pone.0302554 (PMC11065279; doi:10.1371/journal.pone.0302554)
Supplement: Attachment — Submitted filename: Reviewer comments R1.pdf [file pone.0302554.s007.pdf]

Knowledge of female genital schistosomiasis and urinary schistosomiasis among final-year midwifery students in the Volta Region of Ghana.

Thank you for the opportunity to again review the revised paper following comments made by reviewers. The authors should be commended for addressing most of concerns that were raised which has now improved the manuscript. However, there are still some areas that will need to be revised. Below are some outstanding and emerging comments for the authors' consideration:

A. Introduction

1) Lines 110-119 are repeating what has already been reported in lines 87-95 citing the same Nigerian study [13].
2) Line 126-127 – The sentence "It is therefore important to assess the knowledge of health care providers, including those still in training, regarding FGS." should be moved to the next paragraph at the beginning of line 138.
3) Lines 139-141 – authors write "The Volta region was chosen because, historically, communities along the Volta Lake have been known to be endemic for urinary schistosomiasis [15, 27]." Since the purpose of this study was to assess the acquired knowledge/training about US and FGS among midwifery students it is not relevant whether the chosen area was to be an endemic for schistosomiasis or not.

B. Methodology

4) [A previous comment]: Authors tend to merge several sub-sections into one making it difficult for readers to follow the write up. Consider breaking down the sub-heading into separate sub-sections i.e. Study design; Study area; Study population to make it easier for readers to follow the presentation. This comment remains unresolved. For example, in the revised manuscript, authors have chosen to merge 'study design with population' yet the content therein have elements of time and place of the study not related to the subheading. There are so many instances of repetitions within the subheadings, the authors are therefore invited to critically re-examine each sub-heading and the contents to ensure that they are not repeated, easy and logical for a reader to follow.
5) Study site description – authors are presenting data from OPD or DHMIS2 probably to justify why the areas were selected for the study. This again raises the question in 3 above, what have these statistics to do with choice of the midwifery students who were the study population as opposed to community members whose data was recorded at OPD/DHMIS2?
6) Sample size estimation should be preceded by a 'study population description' sub-section, either separately or merged.
7) Study procedures and data collection – line 184 authors write "All final-year midwifery students in the Volta region were included in the study." This sentence is contradictory with what is immediately written up to line 197.

B. Discussions

8) Since the authors indicated that in Ghana only US unlike FGS is part of curricula in medical training institutions. The authors need to explore and discuss the obtained result in the

present study that indicated schools/workshops as the main source of information about US (54.9%) and FGS (59%) for many of the midwifery students that were interviewed. Were these schools/workshops where they got information at the same training colleges they were enrolled at the time of the study or different ones since these are not yet qualified midwives?

9) In lines 347-350 authors write "Participants from Keta NMTC had the highest proportion of participants who had heard about urinary schistosomiasis followed by UHAS and Hohoe NMTC respectively. This could be because Keta has a lot more water bodies than the other places and possibly a higher burden of urinary schistosomiasis." Can authors explain the connection between the environmental setting of the area with the respondent midwifery students' acquired knowledge/training in relation to comments 3 and 5 above?

10) Another limitation of the study is that it was conducted among pre-service students, hindering its generalizability to the in-service group of health worker cadres who acquire more exposure and experience while interacting with community members than the students who are mostly attending lessons in their colleges.

C. Conclusions

11) The first sentence should qualify "[…] among final year midwifery students in the Volta Region, Ghana".
