# Supplementary material for: Knowledge of female genital schistosomiasis and urinary schistosomiasis among final-year midwifery students in the Volta Region of Ghana
Source: PLoS One. 2024 May 2;19(5):e0302554. doi: 10.1371/journal.pone.0302554 (PMC11065279; doi:10.1371/journal.pone.0302554)
Supplement: Attachment — Submitted filename: Reviewer comments R2.pdf [file pone.0302554.s009.pdf]

Knowledge of female genital schistosomiasis and urinary schistosomiasis among final-year midwifery students in the Volta Region of Ghana.

Thank you for the opportunity to re-review the revised manuscript following comments I made. The authors have now addressed all the concerns that were raised which has led to a significant improvement of the manuscript. I have no further comments for the authors.
